# Headache Characteristics of Pediatric Sport-Related Concussion

**DOI:** 10.3390/ijerph21070813

**Published:** 2024-06-21

**Authors:** Michael J. Popovich, Brandon S. Wright, Abigail C. Bretzin, Mark T. Roberts, Bara Alsalaheen, Andrea A. Almeida, Matthew T. Lorincz, James T. Eckner

**Affiliations:** 1 Department of Neurology, University of Michigan, Ann Arbor, MI 48109, USA; marrober@med.umich.edu (M.T.R.); exec.dir@betteryouhomehealth.com (B.A.); almeidaa@med.umich.edu (A.A.A.); lorincz@med.umich.edu (M.T.L.); 2 Department of Kinesiology, University of Michigan, Ann Abror, MI 48109, USA; wrigbran@umich.edu; 3 Injury Prevention Center, Department of Emergency Medicine, University of Michigan, Ann Arbor, MI 48109, USA; brabigai@med.umich.edu; 4 Department of Physical Medicine and Rehabilitation, University of Michigan, Ann Arbor, MI 48109, USA; jeckner@med.umich.edu

**Keywords:** concussion, headache, headache after concussion (HAC), post-traumatic headache, migrainous

## Abstract

Background: Headache is among the most common symptoms following concussion, yet headache after concussion (HAC) remains poorly characterized. This study describes headache characteristics over the first four weeks following pediatric sport-related concussion. Methods: This is a retrospective case series of 87 athletes (mean: 14.9 years; range: 8.4–18.8 years; 38% female) treated in a specialty sports concussion clinic within 28 days of injury. Primary outcomes of headache consistency, frequency, duration, and associated migrainous symptoms were assessed at immediate (0 to 48 h) and weekly time points over the first 28 days post-injury. Generalized mixed linear models compared headache characteristics across time points. Secondary analyses compared each outcome by as-needed analgesic use. Results: During the immediate post-injury period, headache was more often constant (*p* = 0.002) and associated with migrainous symptoms (*p* < 0.001). By the third week post-injury, episodic headache was more prevalent (*p* < 0.001). Most patients (54%) transitioned from constant, migrainous headache to episodic, non-migrainous headache. This finding was uninfluenced by as-needed analgesic medication use. Conclusions: These findings document the trajectory of HAC. Future studies should assess relationships between initial headache characteristics and recovery.

## 1. Introduction

Sport-related concussion (SRC) has gained publicity in recent years with increasing participation in youth athletics and an estimated annual incidence of SRC at 3.8 million in the United States [1,2]. Headache is among the most common symptoms experienced following concussion, having been reported to occur in up to 90% of patients after concussion [3,4,5]. Headache has also been shown to be a commonly provoked symptom during exercise in patients following sport-related concussion [6], which may prevent successful completion of a return-to-play progression that is recommended before a return to full physical activity [7,8]. Headache after concussion (HAC) has been reported to often be the last concussion-related symptom to resolve, highlighting its role in extending the length of recovery [9]. The lack of early resolution of headaches also has been associated with prolonged recovery from concussion [10,11]. In turn, headache is a common component of persisting symptoms after concussion, contributing to the long-term morbidity from concussion [12].

As the clinical presentation of post-concussion symptoms does not remain static during typical recovery, it is crucial to understand the typical evolution of common symptoms after concussion [8]. Despite the pervasiveness of HAC, its characteristics during the expected recovery period in pediatric patients have not been well defined in the literature to date. Notably, the commonly studied post-concussion symptom evaluation tools query headache severity [13,14,15,16], but other headache characteristics, such as the consistency, frequency, duration, and associated symptoms of HAC, are not a component of these standardized assessments. However, these characteristics are important to fully understand the burden of headache symptoms. In general, a greater understanding of the typical trajectory of signs and symptoms after concussion carries prognostic utility and also allows for the early identification of clinically addressable issues that may otherwise lead to a prolonged recovery, as has been shown with vestibular and ocular-motor impairments [17,18]. The clinical practice in post-concussion headache treatment is not uniform, with wide variability in the use of medications and other interventions to treat headache and few clinical trials investigating treatments of HAC [19,20]. Given the lack of standardized guidelines on the optimal management for HAC, a more thorough description of the trajectory of headache characteristics during concussion recovery has potential implications to direct future studies on headache treatments.

Additionally, the current diagnostic criteria for HAC may not capture its full attributes in the expected recovery period from concussion. The International Classification of Headache Disorders (ICHD) uses the term post-traumatic headache to describe HAC. However, the current diagnostic criteria for post-traumatic headache is broad and widely defines the timing of HAC within one week to three months of head injury [21]. There has been debate over the proper diagnostic classification of HAC, given their heterogenous presentation [22]. Previous studies have recognized that HAC may resemble primary headache disorders, such as migraine or tension headache, and these are typically presented as distinct and static phenotypes [23,24]. While others have investigated longer-term changes in HAC (e.g., if persisting for ≥3 months after concussion) [4], the characteristics of headache during the expected recovery period following concussion are not well defined, and it has not been described if HAC maintains the same characteristics throughout typical recovery from concussion. A better understanding of the trajectory of headache characteristics following concussion may inform earlier opportunities to address treatable symptoms, indicate recovery progression, and ultimately improve recovery.

The objective of this study was to describe headache characteristics during the first four weeks following pediatric sport-related concussion. We hypothesize that the consistency, frequency, duration, and associated symptoms of HAC will change throughout recovery across the patient sample and within individual patients.

## 2. Materials and Methods

### 2.1. Study Patient Sample

Chart review was performed of new patients seen in the same sport concussion clinic at a tertiary care center from July 2018 through June 2019. Patients were eligible to be included in this study if they were athletes ≤ 18 years old (range: 8.4–18.8 years), were diagnosed with concussion, and were seen for their initial clinic visit within 28 days from the date of injury. A physician with specialized training in the care of sport-related concussion evaluated the patients and made the diagnosis of concussion based on clinical history and physical examination findings, in accordance with the most recent consensus statement guidelines [25]. To maintain consistent documentation, the study patients were all seen by the same physician. As this study was performed as part of a secondary analysis of existing clinical data, the need for additional parental consent was deemed exempt by the institutional IRB.

### 2.2. Data Collection

Patient charts were reviewed, and data were extracted for each clinic visit until the patients completed their care. Charts were systematically reviewed for the following information pertinent to headache at the time of each visit: presence or absence of headache, consistency, frequency, duration, and symptoms associated with headache. The consistency of headache was classified as either constant or episodic. Headache was considered constant if it persisted for >24 h from its onset after concussion, and it persisted without headache-free intervals. Headache was considered episodic if there were discrete headache episodes but with some periods of headache freedom. Episodic headache was further stratified by frequency as occurring daily or occurring less than daily. In addition, episodic headache was classified by duration as lasting greater than one hour (i.e., any discrete headache episode persisted for more than one hour) or lasting less than one hour (i.e., all discrete headache episodes persisted for less than one hour). A symptom was considered associated with headache if it occurred with or worsened in relation to the occurrence of headache, or in the case of a constant headache worsened in relation to increased headache pain. Due to their similarity with the symptoms often associated with migraine headaches [21], these symptoms are hereafter referred to as migrainous symptoms. Associated migrainous symptoms included photophobia, phonophobia, nausea/vomiting, dizziness, light-headedness, and blurred vision. Headaches were classified as resolved if the headache stopped occurring without re-occurrence of the headache, based on the history obtained in the clinic. Patient-reported as-needed analgesic medication use was also extracted from patient medical records. Medications included acetaminophen, ibuprofen, naproxen, and combination of acetaminophen, aspirin, and caffeine (i.e., Excedrin). Similarly, we also analyzed patient sex to determine its influence on our findings.

Both headache characteristics and medication use were categorized based on their proximity to the time of concussion, as follows: immediate post-injury period (i.e., the first 48 h after concussion); post-injury week one (i.e., within days 2–7 post-injury); post-injury week two (i.e., within days 8–14 post-injury); post-injury week three (i.e., within days 15–21 post-injury); post-injury week four (i.e., within days 22–28 post-injury). Headache characteristics within the immediate post-injury period (0–48 h) were recorded as part of the clinical history taken during the first clinic visit (*n* = 87). All subsequent headache characteristics and as-needed analgesic medication use were recorded based on their status (as reported by the patient and/or their parent) at the time of each clinic visit. In addition to these headache characteristics, the patient demographic information, pre-injury baseline information (relevant aspects of medical history), injury, and treatment characteristics listed in Table 1 were extracted from the medical record.

### 2.3. Statistical Analyses

For the primary objective, the occurrences of the headache characteristics corresponding to each post-injury time point for the overall patient sample are listed as counts and frequencies. For each patient in the sample, transitions in headache characteristics (e.g., from initial constant headache to episodic headaches) also are listed as counts and frequencies. To evaluate for differences in the occurrences of the headache consistency, frequency, duration, and associated symptom characteristics throughout the post-injury time points, a series of generalized linear mixed models were used. A headache characteristic classification was used as the outcome variable for each model, with separate models for headache consistency, frequency, duration, and association with migrainous symptoms. The post-injury time points, defined earlier, were set as a fixed effect in the model. Two-tailed *p*-values were obtained, and to account for multiple comparisons, a false discovery rate (FDR) was used to determine statistical significance [26]. As a secondary objective, headache characteristics were analyzed along with the use of medication at each post-injury period. Chi-squared analysis compared headache consistency (constant vs. episodic) and associated symptoms (migrainous vs. non-migrainous) within the sample population that reported use of as-needed analgesic medications. Patient sex was also introduced in a sensitivity analysis to determine the influence of sex on our findings. All statistical analyses were completed using SPSS Statistics, version 27 (IBM, Armonk, NY, USA).

## 3. Results

### 3.1. Headache Characteristics throughout Recovery in the Patient Sample

A total of 112 charts were reviewed, and 87 patients met the inclusion criteria for this study (62.1% male). Full patient sample characteristics are listed in Table 1. Some patients in the sample were not seen in all post-injury time periods which contributed to variability in the number of patients assessed across the time periods. All patients in the sample reported HAC in the immediate post-injury period (i.e., the first 48 h after concussion).

**Figure 1:**
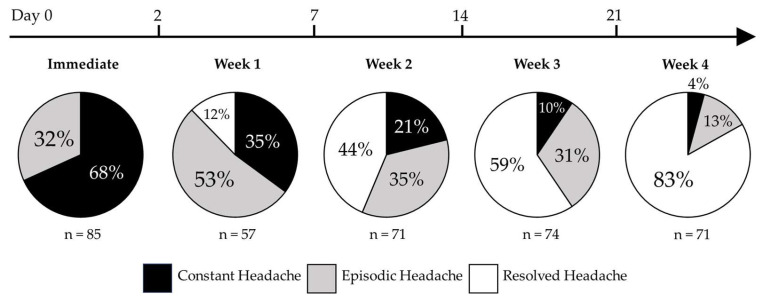
Headache characteristics for patients in the immediate post-injury period through post-injury week four. Headaches were characterized as either constant, episodic, or resolved and converted into percentages of total patients assessed at each of the post-injury time periods.

Table 2 lists the results of the generalized linear mixed models comparing the occurrences of headache consistency from the immediate post-injury period through post-injury week four. Constant headache was more likely than episodic headache in the immediate post-injury period (*p* = 0.002). By post-injury week two, resolved headaches were more likely than constant headaches (*p* = 0.018), and by post-injury week three, episodic headaches also were more likely than constant headaches (*p* < 0.001). Comparisons of specific episodic headache frequency categories (i.e., daily and less than daily) and headache duration categories (i.e., greater than one hour and less than one hour) are included as Table A1 and Table A2 (Appendix A). Figure 1 displays the percentage of the sample that reported constant headache, episodic headache, or no headache in the first four weeks following concussion.

**Figure 2:**
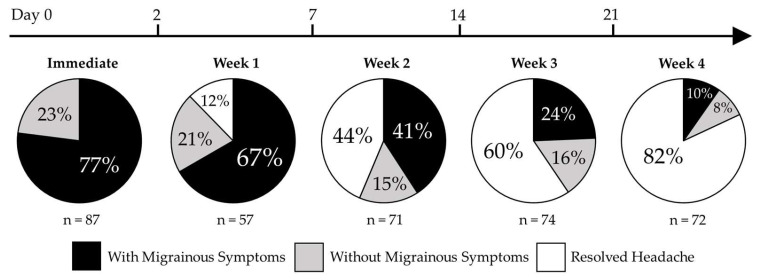
Headache association with and without migrainous symptoms for patients in the immediate post-injury period through post-injury week four. Headaches were designated as either associated with migrainous symptoms, without migrainous symptoms, or resolved and converted into percentages of total patients assessed at each of the post-injury time periods.

Table 3 lists the results of the generalized linear mixed models comparing the occurrences of headaches associated with and without migrainous symptoms at each post-injury time point. Headaches associated with migrainous symptoms were more likely than headaches without associated migrainous symptoms in the immediate post-injury period (*p* < 0.001) and post-injury week one (*p* = 0.001). By post-injury weeks three and four, resolved headaches were more likely than headaches associated with migrainous symptoms and headaches not associated with migrainous symptoms. Figure 2 displays the percentage of the sample that reported a headache associated with migrainous symptoms, a headache without associated migrainous symptoms, or no headache in the first four weeks following concussion.

During sensitivity analysis, males and females did not have significant differences in headache consistency, frequency, duration, and associated migrainous symptoms during all post-injury time periods (*p* > 0.05).

### 3.2. Changes in Headache Characteristics in Individual Patients

In a number of individual patients from the sample, headache characteristics changed from their initial form prior to resolution of headaches. Table 4 lists the number of headache frequency and duration characteristic changes within individual patients. In total, 54% of patients (*n* = 47/87) transitioned from an initial constant headache to episodic headaches prior to resolution of headaches, and in a subset of patients, the frequency and duration of episodic headache changed prior to headache resolution.

A similar transition of the association of headaches with and without migrainous symptoms also occurred in a number of individual patients prior to headache resolution. An amount of 32% of patients (*n* = 28/87) transitioned from headaches associated with migrainous symptoms to headaches without associated migrainous symptoms. An amount of 46% of patients (*n* = 40/87) experienced only headaches associated with migrainous symptoms. An amount of 22% of patients (*n* = 19/87) experienced only headaches without associated migrainous symptoms.

### 3.3. As-Needed Analgesic Use and Headache Characteristics

At post-injury weeks one and two, as-needed analgesic medication use was more likely with headache associated with migrainous symptoms compared to headache without associated migrainous symptoms (*p* < 0.05). Otherwise, there were no significant differences in reported as-needed analgesic medication use based on headache consistency across the post-injury time periods or based on association with or without migrainous symptoms at the immediate post-injury time period or post-injury weeks three or four. Table 5 lists the comparisons of reported as-needed analgesic use by headache consistency and association with or without migrainous symptoms throughout each of the post-injury time periods.

## 4. Discussion

The results of this study demonstrate the evolving characteristics of HAC in a pediatric athlete sample. Most commonly, HAC characteristics transitioned from constant and migrainous to episodic and non-migrainous during the typical recovery period in the pediatric population. The observation that HAC occurred in all patients included in the sample is not surprising, as much work has demonstrated the high incidence of HAC [27]. The frequency and intensity of HAC, which is minimally characterized in the early time period following injury, can be interpreted in relationship to recovery. Our results show that in the immediate period following concussion (i.e., in the first 48 h of concussion), the majority of patients reported constant headache (67%, *n* = 58/87) which was associated with migrainous symptoms (77%, *n* = 67/87), such as photophobia and phonophobia. Only a minority of patients reported episodic headache (31%, *n* = 27/87) or headache without associated migrainous symptoms (23%, *n* = 20/87) in the immediate post-injury period. Overall, there was a trend towards less frequent, shorter-lasting, and less associated migrainous symptoms with headaches prior to recovery. By the fourth week following concussion, most of the total patient sample was no longer experiencing headache (82%), and only a small number of patients in the sample were reporting long-lasting headache (i.e., constant headache or episodic headache lasting greater than one hour).

The results of this study uniquely highlight changes in headache characteristics during the typical recovery period. Based on this study’s findings, it is expected that a concussed patient most likely will experience an initial headache that is constant for at least the first 48 h after concussion and is associated with migrainous symptoms. This is consistent with a previous study showing the predominant form of post-traumatic headache in the early period after injury initially presented as a continuous (i.e., constant) headache with associated symptoms including photophobia, phonophobia, nausea, and vomiting [5]. Another previous study has shown a correlation between post-concussion headache and migrainous symptoms at initial presentation for evaluation [28]. However, by the third week post-concussion, the results of the present study suggest that it is more common to see episodic headaches compared to headache that has remained constant since the time of injury. Similarly, by the third week post-concussion, daily headaches (either constant or episodic) were no longer the predominant headache frequency when compared to less than daily headaches, and by the second week post-concussion, headaches associated with migrainous features were no longer the predominant headache type compared to those without associated migrainous features. While the present study includes only a limited evaluation of associations of medication treatments with headache characteristics, the results do not suggest that as-needed analgesic medication use influenced the reported headache characteristics during recovery. Significantly greater reported as-needed analgesic medication use was only present in headache with associated migrainous symptoms compared to headache without migrainous symptoms. Therefore, there are no findings to suggest that transitions in headache characteristics (e.g., constant to episodic) were explained by the use of analgesic medications. Moreover, our conclusions were not altered when we included sex in our analysis, as there were not any differences among the investigated headache characteristics between males and females.

The observed changes of headache characteristics during recovery in the overall sample also are seen to occur individually within many patients and may highlight HAC’s distinction from other common headache disorders. This study suggests that, for a large portion of patients, HAC may undergo several transformations before resolution: from constant to episodic, from more frequent (i.e., occurring daily) to less frequent (i.e., occurring less than daily), from longer duration (i.e., lasting greater than one hour) to shorter duration (i.e., lasting less than one hour), and from an association with migrainous symptoms to a lack of association with migrainous symptoms. Many patients appear to transition between headache sub-types (e.g., headache associated with migrainous symptoms to headache without associated migrainous symptoms) during the recovery from concussion rather than maintaining one headache type after concussion. These findings challenge previous work that has parallelled HAC with other headache disorders like migraine or tension headache. Specifically, some evidence suggests that concussion and migraine headaches share pathophysiological pathways involving altered ionic flux and increased intracellular/extracellular ionic concentrations [29,30,31]. Although the shared pathophysiology may support the prevalence of headache or migraine as a post-concussion feature, the evolving nature of HAC clinically suggests that it is different from these headache disorders. More recent research has supported this concept mechanistically, showing differences in neuropathological processes, brain structure, inflammation, and head imaging findings in post-traumatic headache compared to migraine [32,33,34]. A study conducted by Chong et al. utilized MRI to find that regional brain structures and fibertracts that play a role in pain processing differentiate the post-traumatic headache and migraine during classification [35]. This clinical study did not investigate the pathophysiological mechanisms underlying headache characteristic evolution after concussion, but this is a potential topic of future exploration. Of note, the headache characteristics in this study were not defined according to diagnostic criteria for primary headache disorders or for post-traumatic headache. In part, this was carried out to facilitate analysis of the available data; for instance, as the available data did not allow for more specific identification of headache duration beyond the classifications used (i.e., greater than or less than one hour), further delineation of these characteristics was not possible. Additionally, while HAC can resemble a primary headache-type such as migraine or tension headache, these were viewed as a different entity than these primary headache disorders, and so they were not strictly defined based on established diagnostic criteria for primary headache disorders.

This study suggests that the characteristics of headache within the first four weeks after concussion evolve in distinct patterns that are not captured by the current diagnostic criteria, which notably do not include the concept that headache characteristic changes may be expected to occur during recovery from head injury [21]. For example, based on the current literature, it is unclear whether HAC is initially constant and directly resolves from this state, or if HAC is initially episodic and directly resolves, or if characteristics of HAC take on a different trajectory during recovery. Based on the results of our study, it may be more useful to approach HAC based on the timing and description of headache at the evaluation, rather than maintaining a strict headache sub-type classification. This study can be utilized as preliminary data to guide improved research that investigates the characterization of HAC. Improved characterization of HAC can help practitioners understand typical progression towards recovery, as well as deviations from this progression that may warrant more conservative or aggressive treatments. Perhaps the most common example based on the present study is a patient reporting constant and/or migrainous headaches beyond two weeks post-injury. In this instance, clinicians may consider more aggressive treatment (e.g., starting a headache preventive medication) to avoid entering a state of prolonged recovery (i.e., post-concussion syndrome). Identifying patterns in HAC characterization may also inform optimal timing or earlier instances to intervene to initiate treatment to reduce recovery time and symptom burden. Future, larger scale studies can use these findings as preliminary data to guide studies on earlier intervention on patients to determine if increasing treatment improves recovery.

There are several additional limitations to this study. These data were obtained from a retrospective chart review, and although efforts were made to maintain consistent documentation of the headache characteristics studied, it is possible that the data were not recorded in a way that fully captured the true evolution of HAC. For example, since headache characteristic data were collected at the time of clinic visits beyond the immediate time period, any headache changes that occurred between visits may not be optimally captured in this study. As a result, the transition of headache characteristics (e.g., associated migrainous symptoms) may be under-reported and may be more common than this study’s results suggest. Relatedly, there was a large amount of missing data pertaining to patient-reported medication use, especially in the immediate post-injury period. For this reason, the primary results regarding headache characteristics and secondary results regarding analgesic use do not align in terms of the number of patients within each time period. Furthermore, this study did not consider the use of preventative headache medications taken prior to injury nor the use of non-pharmacological therapies. Similarly, pertinent concurrent medical history, including depression, anxiety, and ADHD, was collected for the study’s sample to provide a context of potentially important comorbidities that may impact HAC, but analyses regarding these comorbidities was not performed. Future studies could further evaluate for associations between these comorbidities and the characteristics of HAC. Additionally, relevant patient information such as family history of migraines or headaches and sports position during injury were not reported on but could have impacted our findings. This study was performed on a sample of pediatric and adolescent athletes and may not be applicable to older or non-athlete patient populations. The results of this study also may not be applicable to a patient population that is not seen longitudinally in a sport concussion specialty clinic throughout the first month following concussion.

## 5. Conclusions

Headache after sport-related concussion is ubiquitous in youth athletes, and in the immediate 48 h period following sport-related concussion is commonly constant and associated with migrainous symptoms. Over the typical recovery period from concussion, headache that is constant, daily, longer duration, and associated with migrainous symptoms is less often reported, and headache that is episodic, less than daily, short duration, and not associated with migrainous symptoms is more commonly reported, resulting in many patients undergoing a transformation between headache types throughout recovery. Consideration of patient-reported as-needed analgesic medication use did not change this conclusion. Future studies may use headache characteristic information for prognostication of recovery and to determine the optimal timing of headache treatments for persistent headache after concussion.

## Figures and Tables

**Table 1:** Descriptive statistics of the patient sample.

	Frequency (%)	Mean (SD)
Age (years)		14.9 (2.1)
Male	54 (62%)	
History of		
Previous concussion	37 (43%)	
Migraine	17 (20%)	
Depression	6 (7%)	
Anxiety	9 (10%)	
ADHD	15 (17%)	
Days to first clinic visit		7.5 (4.8)
Number of total clinic visits assessed		
1	8 (9%)	
2	45 (52%)	
3	29 (33%)	
4	5 (6%)	
SCAT Symptom Number		9.8 (7.0)
Referred for		
Cervical physical therapy (days)	27 (31%)	15.8 (10.2)
Vestibular physical therapy (days)	33 (37.9%)	15.6 (9.9)
Started on headache preventative medication (days)	4 (4.6%)	33 (10.7)
Reported as-needed headache treatment use		
Immediate post-injury period	61 (71%)	
Entire post-injury period	65 (74.7%)	
Days from concussion until clearance (to complete return-to-play protocol)		29.7 (25.2)
Sport related to concussion		
Football	25 (28.7%)	
Ice Hockey	16 (18.4%)	
Soccer	11 (12.6%)	
Basketball	7 (8.0%)	
Cheerleading	7 (8.0%)	
Lacrosse	4 (4.6%)	
Field Hockey	3 (3.4%)	
Track and Field/Cross Country	2 (2.3%)	
Water Polo	2 (2.3%)	
Martial Arts, Tennis, Swimming, Volleyball, and Wrestling	5 (5.7%)	
Other	5 (5.7%)	

ADHD = Attention-deficit/hyperactivity disorder; SCAT 5 = Sport Concussion Assessment Tool (Version 5).

**Table 2:** Comparisons of headache consistency (constant vs. episodic) at each post-injury time point.

HA Classification Comparison	Post-Injury Time Period	Coefficient Value	Standard Error	FDR Critical Value	*p*-Value
Constant vs. Episodic	Immediate	0.91	0.29	0.023	0.002 *
	Week 1	−0.45	0.37	0.05	0.22
	Week 2	−0.81	0.40	0.035	0.044
	Week 3	−1.79	0.50	0.016	<0.001 *
	Week 4	−1.54	0.74	0.031	0.039
Constant vs. Resolved	Immediate	-	-	-	-
	Week 1	1.02	0.52	0.039	0.052
	Week 2	−1.00	0.42	0.027	0.018 *
	Week 3	−2.42	0.51	0.0077	<0.001 *
	Week 4	−3.22	0.69	0.0039	<0.001 *
Episodic vs. Resolved	Immediate	-	-	-	-
	Week 1	1.48	0.44	0.019	0.001 *
	Week 2	−0.17	0.30	0.043	0.056
	Week 3	−0.53	0.29	0.047	0.070
	Week 4	−1.67	0.39	0.012	<0.001 *

HA = Headache. FDR = False Discovery Rate. * = Reached Statistical Significance Below the FDR Critical Value.

**Table 3:** Comparisons of the occurrences of association with migrainous symptoms at each post-injury time point.

HA Classification Comparison	Post-Injury Time Period	Coefficient Value	Standard Error	FDR Critical Value	*p*-Value
With Migrainous Symptoms vs. Without Migrainous Symptoms	Immediate	1.66	0.35	0.016	<0.001 *
	Week 1	1.52	0.44	0.019	0.001 *
	Week 2	0.77	0.44	0.035	0.078
	Week 3	0.050	0.47	0.05	0.92
	Week 4	−0.33	0.66	0.043	0.62
With Migrainous Symptoms vs. Resolved	Immediate	-	-	-	-
	Week 1	2.18	0.52	0.012	<0.001*
	Week 2	−0.11	0.39	0.047	0.77
	Week 3	−1.20	0.41	0.027	0.004 *
	Week 4	−2.48	0.51	0.0039	<0.001 *
Without Migrainous Symptoms vs. Resolved	Immediate	-	-	-	-
	Week 1	0.39	0.54	0.039	0.47
	Week 2	−1.05	0.40	0.031	0.008 *
	Week 3	−1.30	0.39	0.023	0.001 *
	Week 4	−2.21	0.51	0.0077	<0.001 *

HA = Headache. FDR = False Discovery Rate. * = Reached Statistical Significance Below the FDR Critical Value.

**Table 4:** Headache consistency, frequency, and duration characteristic transitions within individual patients during recovery.

Headache Characteristic Transition	Frequency (%)
Constant only	8 (9%)
Constant → Episodic	47 (54%)
By frequency category	
Constant → daily → less than daily	12 (14%)
Constant → daily	24 (28%)
Constant → less than daily	11 (13%)
By duration category ^a^	
Constant → greater than one hour → less than one hour	4 (5%)
Constant → greater than one hour	22 (25%)
Constant → less than one hour	17 (20%)
Episodic only	32 (37%)
By frequency category ^b^	
Daily → less than daily	7 (8%)
Daily only	22 (25%)
Less than daily only	2 (2%)
By duration category ^c^	
Greater than one hour → less than one hour	6 (7%)
Greater than one hour only	12 (14%)
Less than one hour only	5 (6%)

^a^ Data for headache duration were missing for four patients whose headaches transitioned from constant to episodic. ^b^ Data for headache frequency were missing for one patient who only experienced episodic HAC. ^c^ Data for headache duration were missing for nine patients who experienced only episodic HAC.

**Table 5:** As-needed analgesic medication use and headache characteristics by post-injury time-period. ‘Yes’ indicated positive analgesic use and ‘No’ indicated negative analgesic use.

	Post-Injury Time Period				
PRN Medication Use	Immediate (*n* = 9)	Week 1 (*n* = 45)	Week 2 (*n* = 69)	Week 3 (*n* = 50)	Week 4 (*n* = 29)
Yes	7 (78%)	16 (36%)	21 (30%)	15 (30%)	3 (10%)
HA Consistency:					
Constant	3 (33%)	8 (18%)	8 (12%)	6 (12%)	2 (7%)
Episodic	4 (44%)	8 (18%)	13 (19%)	9 (18%)	1 (3%)
Associated Symptoms:					
Migrainous	5 (56%)	16 (36%) *	17 (25%) *	11 (22%)	2 (7%)
Non-Migrainous	2 (22%)	0 (0%)	4 (6%)	4 (8%)	1 (3%)
No	2 (22%)	29 (64%)	48 (70%) *	35 (70%) *	26 (90%) *

* Reached statistical significance (chi-squared or Fischer test) compared to opposing group (yes vs. no, constant vs. episodic, migrainous vs. non-migrainous; *p* < 0.05).

## Data Availability

The raw data supporting the conclusions of this article will be made available by the corresponding author (M.J.P.) upon request.

## Appendix A

**Table A1:** Comparisons of the occurrences of headache frequency (daily constant, daily episodic, less than daily episodic) at each post-injury time point.

HA Classification Comparison	Post-Injury Time Period	Coefficient Value	Standard Error	FDR Critical Value	*p*-Value
Daily Constant vs. Daily Episodic	Immediate	1.02	0.30	0.021	0.001 *
	Week 1	−0.30	0.38	0.048	0.43
	Week 2	−0.53	0.41	0.038	0.20
	Week 3	−1.02	0.55	0.034	0.067
	Week 4	−0.94	0.80	0.040	0.24
Daily Constant vs. Less Than Daily Episodic	Immediate	3.07	0.62	0.0038	<0.001 *
	Week 1	1.46	0.59	0.025	0.014 *
	Week 2	0.61	0.53	0.042	0.25
	Week 3	−1.15	0.54	0.030	0.032
	Week 4	−0.73	0.82	0.046	0.38
Daily Constant vs. Resolved	Immediate	-	-	-	-
	Week 1	0.99	0.52	0.032	0.058
	Week 2	−0.98	0.41	0.027	0.018 *
	Week 3	−2.38	0.50	0.0076	<0.001 *
	Week 4	−3.18	0.68	0.0019	<0.001 *
Daily Episodic vs. Less Than Daily Episodic	Immediate	2.03	0.62	0.017	0.001 *
	Week 1	1.77	0.55	0.019	0.001 *
	Week 2	1.10	0.48	0.029	0.021 *
	Week 3	−0.17	0.44	0.049	0.69
	Week 4	0.15	0.68	0.05	0.82
Daily Episodic vs. Resolved	Immediate	-	-	-	-
	Week 1	1.28	0.45	0.023	0.005 *
	Week 2	−0.49	0.32	0.036	0.13
	Week 3	−1.33	0.38	0.013	<0.001 *
	Week 4	−2.30	0.49	0.0095	<0.001 *
Less Than Daily Episodic vs. Resolved	Immediate	-	-	-	-
	Week 1	−0.58	0.65	0.044	0.37
	Week 2	−1.66	0.47	0.011	<0.001 *
	Week 3	−1.27	0.36	0.015	<0.001 *
	Week 4	−2.53	0.54	0.0057	<0.001 *

HA = Headache. FDR = False Discovery Rate. * = Reached Statistical Significance Below the FDR Critical Value.

**Table A2:** Comparisons of the occurrences of headache duration at each post-injury time point.

HA Classification Comparison	Post-Injury Time Period	Coefficient Value	Standard Error	FDR Critical Value	*p*-Value
Constant vs. Episodic More Than One Hour	Immediate	1.24	0.32	0.013	<0.001 *
	Week 1	0.14	0.41	0.040	0.74
	Week 2	0.018	0.45	0.048	0.97
	Week 3	−0.91	0.55	0.034	0.10
	Week 4	0.016	0.96	0.049	0.99
Constant vs. Episodic Less Than One Hour	Immediate	3.08	0.62	0.0057	<0.001 *
	Week 1	1.11	0.52	0.025	0.033
	Week 2	0.024	0.45	0.046	0.96
	Week 3	−1.00	0.54	0.030	0.065
	Week 4	−1.22	0.75	0.036	0.11
Constant vs. Resolved	Immediate	-	-	-	-
	Week 1	1.03	0.52	0.029	0.047
	Week 2	−0.95	0.41	0.023	0.019 *
	Week 3	−2.30	0.50	0.0076	<0.001 *
	Week 4	−3.10	0.67	0.0038	<0.001 *
Episodic More Than One Hour vs. Episodic Less Than One Hour	Immediate	1.83	0.62	0.017	0.003 *
	Week 1	0.97	0.48	0.027	0.044
	Week 2	−0.006	0.43	0.05	0.99
	Week 3	−0.10	0.44	0.044	0.82
	Week 4	−1.26	0.80	0.038	0.12
Episodic More Than One Hour vs. Resolved	Immediate	-	-	-	-
	Week 1	0.83	0.46	0.032	0.076
	Week 2	−1.01	0.36	0.021	0.006 *
	Week 3	−1.38	0.36	0.011	<0.001 *
	Week 4	−3.18	0.73	0.0019	<0.001 *
Episodic Less Than One Hour vs. Resolved	Immediate	-	-	-	-
	Week 1	−0.18	0.58	0.042	0.76
	Week 2	−1.05	0.38	0.019	0.006 *
	Week 3	−1.28	0.37	0.015	0.001 *
	Week 4	−1.97	0.43	0.0095	<0.001 *

HA = Headache. FDR = False Discovery Rate. * = Reached Statistical Significance Below the FDR Critical Value.
